# Cerliponase Alfa for the Treatment of Atypical Phenotypes of CLN2 Disease: A Retrospective Case Series

**DOI:** 10.1177/0883073820977997

**Published:** 2020-12-23

**Authors:** Eva Wibbeler, Raymond Wang, Emily de los Reyes, Nicola Specchio, Paul Gissen, Norberto Guelbert, Miriam Nickel, Christoph Schwering, Lenora Lehwald, Marina Trivisano, Laura Lee, Gianni Amato, Jessica Cohen-Pfeffer, Renée Shediac, Fernanda Leal-Pardinas, Angela Schulz

**Affiliations:** 1 37734 University Medical Center Hamburg-Eppendorf, Children’s Hospital, Hamburg, Germany; 2 CHOC Children’s Specialists, Orange, CA, USA; 3 University of California-Irvine School of Medicine, Irvine, CA, USA; 4 2650 Nationwide Children Hospital Columbus Ohio, Ohio State University, Columbus, OH, USA; 5 Bambino Gesu, Rome, Italy; 6 The NIHR Great Ormond Street Hospital, Biomedical Research Centre, London, UK; 7 Hospital de Niños de la Santísima Trinidad [Holy Trinity Children’s Hospital], Cordoba, Argentina; 8 Biostats LLC, Las Vegas, NV, USA; 9 10926 BioMarin, Novato, CA, USA

**Keywords:** neuronal ceroid lipofuscinosis type 2, late infantile neuronal ceroid lipofuscinosis, CLN2 disease, natural history, atypical, cerliponase alfa

## Abstract

**Background:**

The classic phenotype of CLN2 disease (neuronal ceroid lipofuscinosis type 2) typically manifests between ages 2 and 4 years with a predictable clinical course marked by epilepsy, language developmental delay, and rapid psychomotor decline. Atypical phenotypes exhibit variable time of onset, symptomatology, and/or progression. Intracerebroventricular-administered cerliponase alfa (rhTPP1 enzyme) has been shown to stabilize motor and language function loss in patients with classic CLN2 disease, but its impact on individuals with atypical phenotypes has not been described.

**Methods:**

A chart review was conducted of 14 patients (8 male, 6 female) with atypical CLN2 phenotypes who received cerliponase alfa. Pre- and posttreatment CLN2 Clinical Rating Scale Motor and Language (ML) domain scores were compared.

**Results:**

Median age at first presenting symptom was 5.9 years. First reported symptoms were language abnormalities (6 [43%] patients), seizures (4 [29%]), ataxia/language abnormalities (3 [21%]), and ataxia alone (1 [7%]). Median age at diagnosis was 10.8 years. ML score declined before treatment in 13 (93%) patients. Median age at treatment initiation was 11.7 years; treatment duration ranged from 11 to 58 months. From treatment start, ML score remained stable in 11 patients (treatment duration 11-43 months), improved 1 point in 1 patient after 13 months, and declined 1 point in 2 patients after 15 and 58 months, respectively. There were 13 device-related infections in 8 patients (57%) and 10 hypersensitivity reactions in 6 (43%).

**Conclusions:**

Cerliponase alfa is well tolerated and has the potential to stabilize motor and language function in patients with atypical phenotypes of CLN2 disease.

The neuronal ceroid lipofuscinoses (NCLs) are a heterogeneous group of lysosomal storage disorders that are associated with a progressive, neurodegenerative course and characterized by varying degrees and onsets of visual loss, seizures, and cognitive dysfunction.^1^ Historically, NCLs were classified by age of onset, progression of symptomatology, and ultrastructural storage morphology. Recent advances in genomics have resulted in the elucidation of 13 gene loci linked to NCLs and a growing recognition of the phenotypic heterogeneity associated with some of these disorders.^1,2^

Neuronal ceroid lipofuscinosis type 2 (CLN2) disease, also known as late infantile neuronal ceroid lipofuscinosis, is one of the most common NCLs and is caused by pathogenic variants in the *TPP1* gene encoding the lysosomal enzyme tripeptidyl peptidase 1 (TPP1). ^1,3,4^ Reported estimates of incidence range from 0.15 to 0.5 per 100 000 in European countries,^5-7^ and up to 9.0 per 100 000 in Newfoundland^8^ and an estimated prevalence of 0.6 to 0.7 per million in Scandinavia.^9^ Individuals with the classic phenotype of CLN2 disease typically exhibit absence of TPP1 enzymatic activity, present between ages 2 and 4 years with seizures and language delay, and experience rapid deterioration of cognitive and motor skills over a 2- to 3-year period following initial presentation.^10,11^ Visual deterioration usually becomes evident by age 7 years and eventually leads to blindness.^10^ Death typically occurs between age 8 years to midadolescence.^10,11^ However, a subset of TPP1-deficient patients do not follow the classic clinical course. Although documented to have TPP1 enzymatic deficiency and/or *TPP1* variants, individuals with atypical phenotypes are reported to have variable age of onset, variable symptomatology, and/or a protracted disease course.^4,10,12-24^ Documented symptoms have included ataxia, gait disturbances, coordination difficulties, and cognitive regression, but seizures and visual findings are less frequently reported. Notably, seizures are completely absent in some phenotypes, including in several patients originally diagnosed with autosomal recessive spinocerebellar ataxia (type 7; SCAR7).^17,18,24,25^ The literature on the atypical population is currently limited to case reports and case series stemming from single countries or specific geographic regions, and the full spectrum of CLN2 disease has yet to be well characterized.

Cerliponase alfa (Brineura, BioMarin), a recombinant human TPP1 enzyme replacement therapy delivered via intracerebroventricular (ICV) infusion, has been shown to stabilize motor and language function loss in individuals with CLN2 disease.^26^ To date, cerliponase alfa has received approval in several countries. The effect of intracerebroventricular-administered cerliponase alfa therapy on the disease course in atypical patients has not been described. The objectives of this study were to characterize the disease course and assess the impact of cerliponase alfa treatment on individuals with atypical phenotypes of CLN2 disease.

## Methods

This was a retrospective chart review conducted at 6 sites in 5 countries (Germany, United Kingdom, Italy, United States, and Argentina). Medical history data were collected on patients who met the following criteria: (1) had a confirmed diagnosis of CLN2 disease by enzymatic and/or molecular methods, (2) exhibited atypical symptomatology and/or disease progression compared with the classic phenotype, and (3) was receiving treatment with cerliponase alfa. Written informed consent was collected prior to the chart review.

Disease progression was quantitatively assessed for each patient by their treating physician using reported scores from the motor and language (ML) domains of the CLN2 Clinical Rating Scale, an established method to measure the clinical course of classical CLN2 disease and the clinical impact of cerliponase alfa therapy.^10,26,27^ The CLN2 Clinical Rating Scale is based primarily on natural history data from individuals with classical phenotypes. Because of the dearth of extensive natural history data on individuals with atypical presentations, no comparative tool based on disease progression data from atypical phenotypes currently exists; the CLN2 Clinical Rating Scale, which allows both retrospective and prospective natural history data collection, was therefore implemented to evaluate treatment effect in this subset of patients by monitoring changes in language and motor functions The ML score ranges from 0 to 6, with 0 representing complete loss of function, 1 representing severe abnormality, 2 representing slight abnormality, and 3 representing normal function within each of the 2 domains (Table 1). Information about the onset of seizures and vision loss were also collected. Safety data specifically related to cerliponase alfa and the intracerebroventricular infusion device were collected. Some patients participated in a compassionate use programme, and data were collected for monitoring purposes. Descriptive statistics were performed using SAS, version 9.4 (Cary, NC, USA). Pretreatment rates of decline in ML score were calculated as the change from the last recorded ML score of 6 prior to decline to the last recorded ML score prior to start of treatment divided by the time interval between these 2 assessments. These rates were annualized in order to enable direct comparisons between subjects and to facilitate a comprehensive analysis of the full cohort.

**Table 1.** CLN2 Clinical Rating Scale (Motor and Language Domains).^27^

	Motor	Language
Score 3	Grossly normal gait, no prominent ataxia, no pathologic falls	Apparently normal language. Intelligible and grossly age-appropriate. No decline noted yet
Score 2	Independent gait, as defined by ability to walk without support for 10 steps. Will have obvious instability and may have intermittent falls	Language has become recognizably abnormal: Some intelligible words may form short sentences to convey concepts, requests, or needs. This score signifies a decline from a previous level of ability (from the individual maximum reached by the child)
Score 1	Requires external assistance to walk or can crawl only	Hardly understandable. Few intelligible words
Score 0	Can no longer walk or crawl	No intelligible words or vocalizations

## Results

### Patient Characteristics Prior to Treatment

Fourteen patients with atypical phenotypes of CLN2 disease, including 1 sibling pair, were identified, of whom 8 (57%) were male and 6 (43%) were female (Table 2). The median age at first presenting disease symptom was 5.9 years (interquartile range [IQR] 5.0-7.3). The first presenting symptom was language abnormalities in 6 patients (43%), seizures in 4 (29%), a combination of ataxia and language abnormalities in 3 (21%), and ataxia alone in 1 (7%). Over the disease course, 14 patients (100%) developed ataxia or motor (gait) disturbances, 12 (86%) developed language abnormalities, 10 (71%) developed seizures, and 1 (7%) developed vision abnormalities.

**Table 2.** Patient Characteristics and Pretreatment ML Scores.

Patient	Sex	First symptom	Age at symptom onset (y)	Age at diagnosis (y)	Time to diagnosis from symptom onset (y)	Other disease symptoms	*TPP1/CLN2* mutations	First recorded pretreatment ML score	Last recorded pretreatment ML score	Annualized rate of decline in ML score prior to treatment
1	M	Ataxia/motor disturbance, language regression	10	11	1.4	None	c.622C>T, c.38T>C	6	4	1.0
2	F	Ataxia/motor disturbance, language regression	8	9	1.2	Seizures, vision abnormality	c509-1G>C, c.133-1344dup	6	4	0.7
3	F	Ataxia/motor disturbance	6	12	5.6	Language regression, seizures	c.622C>T, c.1340G>A	6	3	0.5
4	M	Ataxia/motor disturbance, language regression	5	8	3.3	Seizures	1340G>A, c1261T>A	6	2	0.8
5	M	Seizures	6	6	0.1	Ataxia/motor disturbance	c.622C>T, c1261T>A	6	6	0.0^a^
6^b^	F	Language abnormalities/regression	8	11	3.0	Ataxia/motor disturbance, seizures	c.622C>T, c887-10A>G	6	3	0.7
7^c^	F	Language abnormalities^d^/regression	3	11	7.5	Ataxia/motor disturbance	c.509-1G>C, 1029G>C	6	3	0.6
8^c^	M	Language regression^e^	8	7	−0.6	Ataxia/motor disturbance	c.509-1G>C, 1029G>C	6	5	0.7^a^
9	M	Seizures	5	14	8.7	Ataxia/motor disturbance, language regression	c.509-1G>C, 1058C>A	6	4	0.3
10	F	Language abnormalities^f^	6	13	6.5	Ataxia/motor disturbance	1340G>A, 89+5 G<C	6	4	0.2
11	M	Seizures	7	11	4.3	Ataxia/motor disturbance, language regression	c.509-1G>C, c.139C>G	6	4	0.4
12	F	Language abnormalities^g^	5	16	11.0	Ataxia/motor disturbance, seizures, language regression	c.1015C>T, c.959T>G	6	3	0.3
13	M	Seizures	5	13	8.0	Ataxia/motor disturbance	c.622C>T, 782T>C	6	3	0.3
14	M	Language regression	5	7	2.8	Ataxia/motor disturbance, seizures	c380G>A, c.1379G>A	6	2	1.1

Abbreviations: CLN2, neuronal ceroid lipofuscinosis type 2; F, female; M, male; ML, motor and language; TPP1, tripeptidyl peptidase 1.

^a^ ML rates of decline for patients 5 and 8 were adjusted to account for the fact that the time interval was less than a year between first recorded ML score and ML score at treatment initiation.

^b^ Language abnormalities include bradylalia, dysphonia, and bradypsychia.

^c^ Patients 7 and 8 are a sibling pair; patient 8 is the younger sibling.

^d^ Language abnormalities include stuttering.

^e^ This patient’s first symptoms also included apraxia.

^f^ Language abnormalities include selective mutism.

^g^ Language abnormalities include delay and regression. Delay was initially reported and attributed to the child being adopted and learning 2 languages. Patient subsequently experienced language regression.

The median age at diagnosis was 10.8 years (IQR 8.6-12.4) (Table 2). The median time between onset of first presenting symptom and diagnosis was 3.8 years (IQR 1.8-7.3); patient 5, a younger sibling of an affected child with an atypical phenotype, was diagnosed prior to developing any disease symptoms. The most common pathogenic variants in this cohort were c509-1G>C, present in 5 patients on 1 allele only, and c.622C>T, present in another 5 patients on 1 allele only. Four patients had neither mutation.

**Figure 1.**
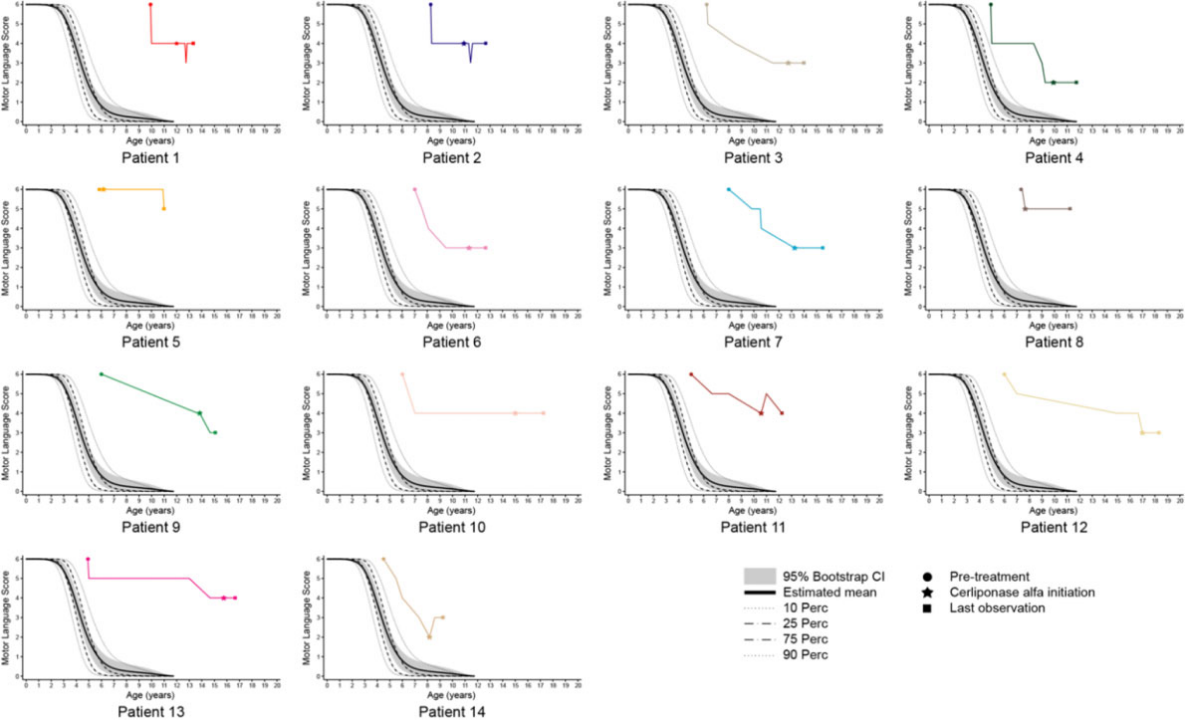
Patient pretreatment and posttreatment ML scores over time. All available motor and language (ML) scores for each patient are shown and underscore the clinical heterogeneity of CLN2 disease. ML scores at 3 time points are marked for each patient: last pretreatment ML score, ML score at cerliponase alfa initiation, and last posttreatment ML score. The classic CLN2 disease course is superimposed.^11^ CI, confidence interval; Perc, percent.

At the time of first assessment, the ML domain score was 6 (normal) for all patients (Table 2, Figure 1). However, 13 patients (93%) experienced a decline in ML score prior to initiation of cerliponase alfa treatment. Patient 5, a younger sibling of an affected child, was started on treatment prior to any symptoms. The median annualized rate of ML score decline during the pretreatment period was 0.5 units/y (range 0.0-1.1 units/y).

### Cerliponase Alfa Treatment

The median age at initiation of cerliponase alfa therapy was 11.7 years (IQR 10.1-13.7). The median treatment duration was 1.5 years (IQR 1.3-2.2; range 11-58 months) (Table 3). The ML score remained stable in 11 patients (treatment duration 11-43 months), improved in 1 patient by 1 point (patient 14 in the motor domain) after 13 months of treatment, and declined in 2 patients by 1 point (patients 5 and 9 in the motor domain) after 15 and 58 months of treatment, respectively (Table 3, Figure 1).

**Table 3.** Posttreatment ML Scores.

Patient	Age at initiation of cerliponase alfa treatment (y)	Treatment duration (y)	ML score at initiation of treatment	Last recorded posttreatment ML score	Change in ML score posttreatment
1	12.0	1.3	4	4	0
2	11.1	1.9	4	4	0
3	12.8	1.3	3	3	0
4	9.9	1.8	2	2	0
5	6.2	4.8	6	5	−1
6	11.3	1.3	3	3	0
7	13.3	2.3	3	3	0
8	7.7	3.6	5	5	0
9	13.8	1.3	4	3	−1
10	15.0	2.3	4	4	0
11	10.6	1.7	4	4	0
12	17.0	1.3	3	3	0
13	15.8	0.9	3	3	0
14	8.2	1.1	2	3	1

Abbreviation: ML, motor and language.

For the purposes of this study, we focused on collecting device-related adverse events and hypersensitivity adverse events considered to be drug related, which are the primary adverse events associated with cerliponase alfa clinical trials.^26^ Device-related adverse events were the most common and included colonizations, which are continuous, asymptomatic, and may or may not require antibiotic treatment, as well as infections, which require treatment. *Cutibacterium acnes* (*Propionibacterium acnes*) colonizations were reported for 7 patients. There were 13 device-related infections (*C acnes* [n=11], *Staphylococcus epidermidis* [n=1], and *Staphylococcus capitis* [n=1]) in 8 patients. There were 2 reports of occlusions in 2 patients. In total, there were 8 port replacements in 6 patients owing to infections or occlusion. Other device-related events were needle dislodgement on 2 occasions in one patient and 1 cerebral hemorrhage following a catheter replacement in another patient. According to label instructions, all patients received regular treatment with an antihistamine to prevent allergic reactions. There were 10 hypersensitivity reactions in 6 patients (43%), including fever, vomiting, tachycardia, and headache, and treatment for these events included prednisolone, ibuprofen, and paracetamol. One patient experienced only fever in 2 events.

## Discussion

Classic late-infantile CLN2 disease is characterized by symptom onset between 2 and 4 years of age and a predictable clinical course marked by epilepsy and rapid psychomotor decline.^10,11^ In an observational study of individuals with CLN2 disease, the median age at symptom onset was 2.9 years, and the most common presenting symptoms were seizures (70%), language abnormalities (delay and/or regression) (57%), and motor difficulty (41%).^11^ The rate of ML score decline for this natural history cohort was 1.8 units/y.^11^ In contrast, the patients in our cohort exhibited delayed onset of disease symptoms (median age at symptom onset was 5.9 years) and slower rates of decline with notable absence of epilepsy in 4 patients (29%). Language regression was the most common presenting symptom (reported in 9 patients [64%]), followed by ataxia/movement difficulty (4 patients [29%]) and seizures (4 patients [29%]). Ataxia/movement disturbance developed in all patients (100%), which is consistent with the literature on atypical populations.^12-15^ Disease progression before treatment was slower compared with classic CLN2 disease (median annualized rate of ML score decline of 0.5 units/y vs 1.8 units/y in untreated patients).^11^

The exclusive presence of the 2 most common pathogenic variants in CLN2 disease, c509-1G>C and c.622C>T, is associated with the classic disease course.^10^ None of our patients were homozygous for one of these alleles, and nor were they compound heterozygous for both alleles. Five (35.7%) were compound heterozygous for c509-1G>C and an uncommon variant, 5 (35.7%) were compound heterozygous for c.622C>T and an uncommon variant, and 4 patients had 2 uncommon variants. The variant c887-10A>G identified in patient 6 has been associated only with atypical phenotypes and only in Spain, Portugal, Chile, Colombia, and Argentina,^28^ where this patient was from. All pathogenic variants identified in our cohort have been reported in the University College London NCL database (CLN2/TPP1 Mutation and Patient Databases; available at https://www.ucl.ac.uk/ncl/CLN2mutationtable.htm, accessed 18 November 2019), with the exception of the c.1333_1344dup variant in patient 2 and the c.782T>C mutation in patient 13, which are only reported in ClinVar (https://www.ncbi.nlm.nih.gov/clinvar/47783801, accessed 18 November 2019).

Open-label studies have demonstrated that intracerebroventricular infusion of 300 mg cerliponase alfa every 2 weeks for 96 weeks slowed deterioration in motor and language function in children with CLN2 disease, as measured by a lower mean (standard deviation) rate of ML score decline compared with untreated controls (0.38 ± 0.101 vs 2.06 ± 0.149 per 48 weeks; *P* < .0001).^26^ In our cohort, treatment with cerliponase alfa over a relatively short assessment period stabilized the ML score in 11 patients (79%) and improved the score in 1 patient (7%) (patient 14), suggesting that cerliponase alfa has the potential to slow disease progression in patients with atypical phenotypes of CLN2 disease. As neuronal loss is irreversible, the gain in ML score experienced by patient 14 is likely attributed to this patient’s recovery from intracerebroventricular device implantation (an event that had prompted a 1-point decline in ML score).

The observed safety profile of cerliponase alfa in this study was consistent with findings described previously for treated patients with classic CLN2 disease.^26^ Age may have influenced the rate of *C acnes* infections in this cohort, as acne is more frequent in adolescents owing to increased sebum production. It has been suggested that bacterial biofilms penetrate into the sebum and act like an adhesive, leading to increased cohesiveness of corneocytes and the formation of microcomedones.^29^ Additionally, a high availability of sebum, a nutritional substrate for *C acnes*, may result in an increased proportion of metabolically active bacteria and contribute to a pro-inflammatory phenotype of the *C acnes* biofilm.^30^ Other common skin pathogens (*S epidermidis* and *S capitis*) were also found.

Diagnosis of CLN2 disease is often delayed owing to its rarity and nonspecific presenting symptoms.^11^ Our findings suggest that the diagnosis of individuals with atypical presentations is even more challenging. Compared with the natural history cohort,^11^ our cohort was diagnosed at a much older age (10.8 years vs 4.5 years), and the median time to diagnosis from initial symptom onset was significantly longer (3.8 vs 1.9 years). Early diagnosis facilitates early initiation of disease-specific care and is critical for family planning. Whereas *TPP1* is currently incorporated in the majority of comprehensive epilepsy gene panels, our study findings underscore the importance of including *TPP1* on ataxia and developmental delay (which include language and motor components) gene panels as well.

There are several limitations associated with this study. The small and variable patient cohort limits the generalizability of the study findings. For the period prior to diagnosis, the collection of medical records for some patients was incomplete owing to multiple referrals. The CLN2 rating scale is a crude measurement tool that provides limited information, and although it is disease specific, it is based primarily on disease progression data from individuals with the classic phenotype; moreover, it was not administered at consistent time intervals for all patients. In the absence of a comparable tool specific for the atypical population, longer follow-up and additional quantitative assessments, such as neurocognitive testing and brain imaging, will be necessary to elucidate the impact of cerliponase alfa therapy on our atypical cohort. Longitudinal observational and interventional studies of atypical CLN2 patients are warranted given the dearth of data on this subpopulation. Nonetheless, these findings drawn from this small cohort suggest that intracerebroventricular-administered cerliponase alfa therapy has the potential to stabilize or slow disease progression in individuals with atypical phenotypes of CLN2 disease.

## Conclusions

Although atypical phenotypes are associated with delayed symptom onset and/or protracted disease course, all patients in our cohort experienced neurodegeneration, as demonstrated by a reduction in ML score. Over the relatively short time period during which patients in this cohort were treated with cerliponase alfa, most had no observed change in their ML scores. Intracerebroventricular cerliponase alfa, the first approved treatment for CLN2 disease, is well tolerated and may have the potential to slow the decline of motor and language function in patients with atypical phenotypes. Longer follow-up will be needed to better understand the clinical impact of this therapy on individuals with atypical presentations of CLN2 disease.
